# Point-of-care ultrasound training among anesthesiology residency programs in the United States

**DOI:** 10.1186/s12871-025-02929-y

**Published:** 2025-02-26

**Authors:** Jordon Edwards, Daniel Ahn, Daniel Alcaraz, Collin Chiles, Tina Khuu, Nilam J. Soni, Varun Goyal, Crystal Manohar

**Affiliations:** 1 Department of Anesthesiology, University of Texas Health Science Center at San Antonio, 7703 Floyd Curl Drive, San Antonio, TX 78229-3900 USA; 2 Long School of Medicine, University of Texas Health San Antonio, San Antonio, TX USA; 3 Department of Medicine, University of Texas Health San Antonio, San Antonio, TX USA; 4 Department of Anesthesiology, University of Arkansas for Medical Sciences, Little Rock, AR USA; 5 Department of Anesthesiology and Pain Medicine, University of California Davis, Sacramento, CA USA

**Keywords:** Ultrasound, Graduate medical education, Anesthesiology, Glucagon-like peptide 1, Focused assessment with sonography for trauma

## Abstract

Point-of-care ultrasound (POCUS) use has become ubiquitous in the field of anesthesiology. However, POCUS training curriculum in anesthesiology residencies vary widely. We performed a survey study to better understand the structure of existing POCUS training and identify barriers to instituting a POCUS curriculum for anesthesiology trainees.

**Supplementary Information:**

The online version contains supplementary material available at 10.1186/s12871-025-02929-y.

## Introduction

Point-of-care ultrasound (POCUS) use is integrated into the bedside evaluation of patients and to aid in real-time clinical decision-making. The Accreditation Council for Graduate Medical Education (ACGME) requires anesthesiology residents to demonstrate competency in knowledge of ultrasound physics, obtaining standard ultrasound views, evaluating organ function and pathology, and guiding procedural techniques [1]. Similarly, the American Board of Anesthesiology (ABA) has updated the Objective Structured Clinical Examination (OSCE) outline to include proficiency in POCUS exams as part of the board certification process [2]. While programs exist to certify trainee competency such as the American Society of Anesthesiology (ASA) Diagnostic POCUS Certificate Program, there is little standardization of training across ACGME-accredited anesthesiology residencies.

## Methods

To understand the structure of existing POCUS training efforts and identify barriers to instituting a standardized POCUS curriculum for trainees, we conducted a survey of anesthesiology residency programs in 2022. The study was approved by the University of Texas Health Science Center at San Antonio Institutional Review Board (protocol number: 22-222E). A 51-item electronic survey (Qualtrics, Provo, UT) was distributed to the program directors of 157 ACGME-accredited anesthesiology residency programs representing all of the programs participating in the Electronic Residency Application Service (ERAS) at the start of our study. The survey was sent to the residency program directors with the option to forward the survey to the appropriate POCUS director or lead faculty within the anesthesiology department. Survey responses were collected over a 4-week period. We sent a reminder message after 2 weeks and also directly contacted program directors to encourage participation. The survey responses were collected, and analyzed.

## Results

**Fig. 1:**
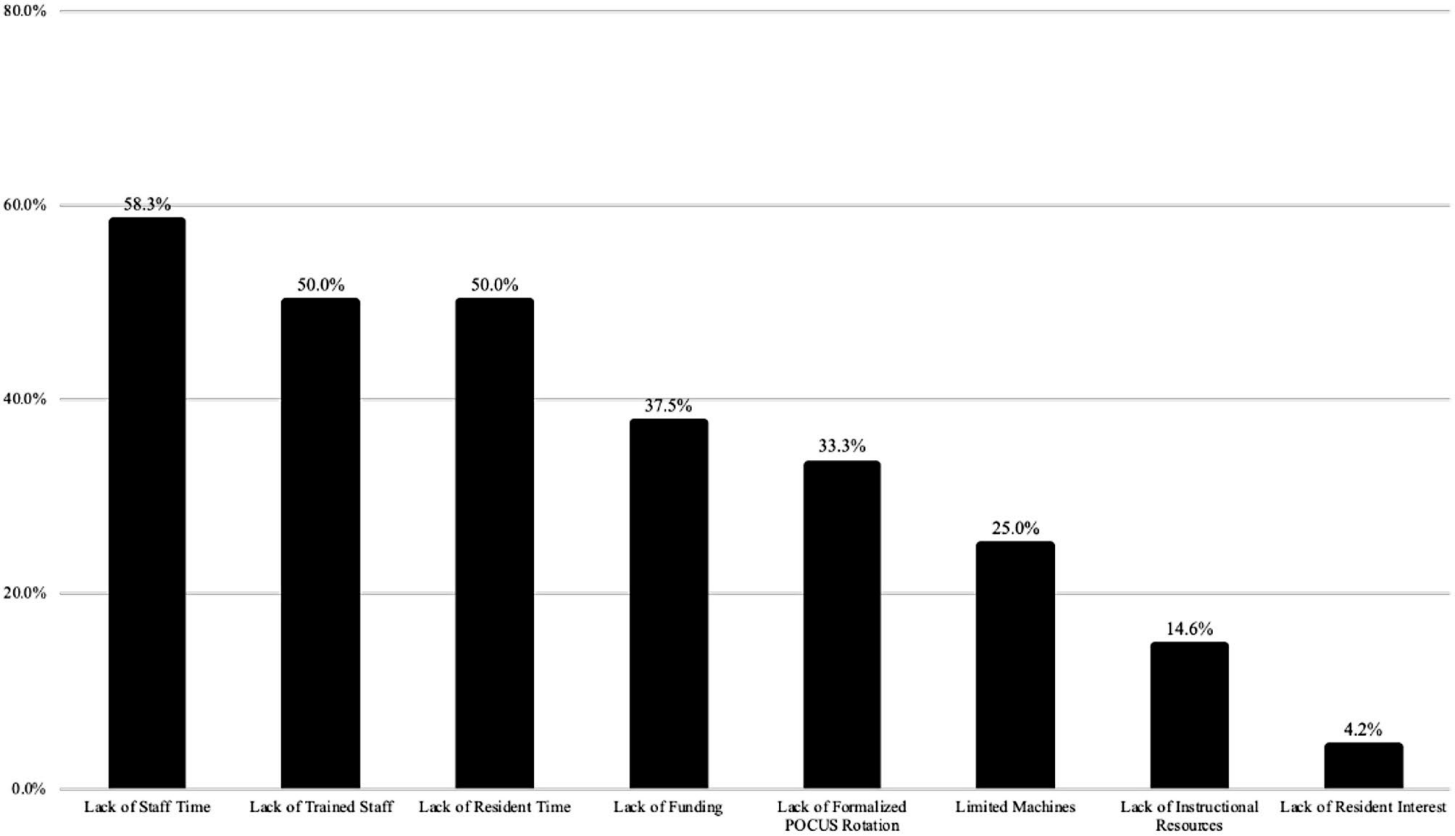
Barriers to Implement POCUS Curriculum

Completed surveys from 48 institutions (a total of 157 institutions) were received from anesthesiology faculty members leading to an overall response rate of 31%. The roles of these faculty members include program directors, associate program directors, POCUS-teaching faculty, and general anesthesiology faculty. A majority of programs used a combination of informal bedside instruction (98%), faculty-led lectures (90%), online modules (77%), and simulation sessions (70%) as methods of POCUS education. Some programs offered mandatory (35%) or elective (50%) POCUS rotations. 60% of programs did not have a formal assessment to test POCUS knowledge or skills of learners. Hands-on skills assessment, formal written exam, ultrasound image or video review, or set number of POCUS scans were used by the programs that offered formal competency assessment. Respondents focused most heavily on the cardiac component of POCUS, with 83% committing an average of > 3 h/year to dedicated cardiac POCUS training. There was a smaller emphasis on lung (63%), gastric (42%), focused assessment with sonography in trauma (FAST) (42%), and airway (21%) ultrasound. Similarly, most programs felt training and competency in cardiac POCUS (67%) was of greatest importance followed by lung (44%), gastric (21%), FAST (25%), and airway (17%) ultrasound exams. Though most programs believed there should be a standardized POCUS curriculum (90%), the lack of trained staff (50%), staff time (58%) and resident time (50%) were the greatest barriers to POCUS training (Fig. 1).

## Discussion

POCUS is a required competency for anesthesiology residents. The 2023 ACGME requirements state that learners must be able to obtain “standard views of the heart, inferior vena cava…allowing the evaluation of myocardial function, estimation of central venous pressure, and gross pericardial/cardiac pathology (e.g. large pericardial effusion).” In addition, the learner must be able to detect pneumothorax and pleural effusions using the ultrasound [1].

Prior survey studies of other medical specialties show somewhat differing uses for POCUS exams. For example, emergency medicine physicians and hospitalists reported frequent use of POCUS for procedural guidance, cardiac, pulmonary, abdominal, musculoskeletal, and deep vein thromboses (DVT) exams [3, 4]. Primary care physicians and rheumatologists performed POCUS mostly for musculoskeletal evaluations and associated procedures (i.e. bursa, joint, tendon injections) though PCPs also reported frequent exams of the bladder to diagnose urinary retention and abscess for drainage [5, 6]. Certain components of POCUS exams from these specialties – such as DVT exams - could provide useful information in the peri-operative setting if incorporated into the anesthesiology training curriculum.

Our results indicate a shortage of trained staff, staff time, and resident time as the primary barriers to incorporating POCUS training into residency, which has been cited in previous studies [3–8]. Among practicing anesthesiologists, lack of training is also the most frequently cited barrier [9]. One solution to this problem is adoption of the ASA Diagnostic POCUS Certificate Program or its methods among faculty [10]. This program pairs learners with local mentors to provide hands-on training and digital educational materials to develop competency in image interpretation/acquisition of the heart, lung, gastric organ systems and the FAST exam. The program is aligned with ABA guidelines regarding POCUS training, utilizes a database of peer-reviewed images, and requires a final written exam for completion. Learners are required to complete 110 image acquisitions (50 cardiac, 30 lung and 30 gastric exams) and 140 interactive cases for interpretation (100 cardiac, 20 lung and 20 gastric exams). This program is costly, but it provides a ready-made curriculum for residents and faculty members who wish to master POCUS and in turn, lead POCUS training at their respective institutions. The ACGME could adopt elements of this program as training.

In addition, the expert panel recommendations from the American Society of Regional Anesthesia and Pain Medicine (ASRA) could serve as another source of guidance [11]. The expert panel recommends a wider range of exams to include 170 image acquisitions (30 airway, 30 lung, 30 FAST, 30 gastric and 50 cardiac exams) and 180 interactive cases for interpretation (20 airway, 20 lung, 20 FAST, 20 gastric, 100 cardiac cases). These could serve as the minimum number of examinations to achieve competence.

Gaining importance is the gastric component of POCUS, which could have an important impact on the perioperative assessment on a subset of patients. These include patients with certain comorbid conditions that cause delayed gastric emptying (i.e. diabetes) and uncertain prandial status (i.e. pregnancy, trauma, pediatrics, and non-English speaker) [12]. Further, patients taking glucagon-like peptide-1 (GLP-1) receptor agonists require special attention. Introduced in 2005, GLP-1 agonists are diabetes and weight loss medications with a half-life of 7 days known to cause delayed gastric emptying. Unsurprisingly, a recent retrospective analysis showed that patients taking GLP-1 agonists had significantly higher residual gastric content compared to control despite appropriate fasting time [13]. The risk of complications due to delayed gastric emptying with the use of GLP-1 agonists remains an area of active research, but with the increasing popularity of these drugs, perioperative gastric ultrasound may gain greater relevance in the assessment of aspiration risk.

A limitation of our study is the low-response rate (31%). Despite efforts to improve responses, including email reminders and in-person flyers distributed at national conferences, the response rate did not improve. In addition, the results are from self-reported data and free-text responses were not available in the survey. Another limitation is a potential bias towards institutions with strong interests in POCUS education and therefore, our results may not accurately reflect the current state of POCUS curricula across the nation.

## Conclusion

Proficiency in POCUS is a critical skill, and a standardized POCUS curriculum is imperative to meet the requirements set forth by the ACGME and ABA. Our study suggests investment of resources to train faculty and allotment of training time for both faculty and residents are important components in this process. Potential solutions include dedicated POCUS rotations integrated in the formal residency curriculum and the ASA Diagnostic POCUS Certificate Program. Our hope is to present this data at national conferences to highlight differences in POCUS curricula for programs across the country. Future work could include updating the survey and re-surveying programs for curriculum updates as well as plans to implement a curriculum if one does not exist.

## Electronic supplementary material

Below is the link to the electronic supplementary material.

Supplementary Material 1

## Data Availability

No datasets were generated or analysed during the current study.

## Abbreviations

POCUS: Point-of-care ultrasound
ACGME: Accreditation council for graduate medical education
ASA: American society of anesthesiology
OSCE: Objective structured clinical examination
ERAS: Electronic residency application service
FAST: Focused assessment with sonography for trauma
GLP-1: Glucagon-like peptide 1
