# Supplementary material for: Point-of-care ultrasound training among anesthesiology residency programs in the United States
Source: BMC Anesthesiol. 2025 Feb 26;25:105. doi: 10.1186/s12871-025-02929-y (PMC11863924; doi:10.1186/s12871-025-02929-y)
Supplement: Supplementary file 1 — Supplementary Material 1 [file 12871_2025_2929_MOESM1_ESM.docx]

Appendix 1. Survey Questions

1. What is the name of your residency program?
2. What state is your residency program located?
3. What is your role in the residency program?
   1. Program/Associate Program Director
   2. Faculty
   3. POCUS Curriculum Director
   4. Other
4. How many total residents are in your program?
5. What training modalities are currently used to teach point-of-care ultrasound examinations? (Select all that apply)
   1. Informal bedside instruction
   2. Online modules
   3. Lectures
   4. Simulation sessions
   5. Structured expert demonstrations
   6. Video/image review
   7. Mandatory ultrasound rotation
   8. Elective ultrasound rotation
   9. Extracurricular course
6. Is dedicated funding available for ultrasound education?
   1. Yes
   2. No
7. From what source is dedicated funding available? (Select all that apply)
   1. Affiliated medical school
   2. Anesthesia department
   3. Sponsor (medical device company)
   4. Other
8. How many total hours per year are dedicated to teaching the following exams? Please include lectures, demonstrations, hands-on sessions, etc.
   1. Heart
      1. 0 hours
      2. 1-2 hours
      3. 3-5 hours
      4. 5-10 hours
      5. 10 or more hours
   2. Lung
      1. 0 hours
      2. 1-2 hours
      3. 3-5 hours
      4. 5-10 hours
      5. 10 or more hours
   3. Gastric
      1. 0 hours
      2. 1-2 hours
      3. 3-5 hours
      4. 5-10 hours
      5. 10 or more hours
   4. FAST
      1. 0 hours
      2. 1-2 hours
      3. 3-5 hours
      4. 5-10 hours
      5. 10 or more hours
   5. Airway
      1. 0 hours
      2. 1-2 hours
      3. 3-5 hours
      4. 5-10 hours
      5. 10 or more hours
9. Is there a minimum number of scans required for:
   1. Heart exams
      1. Yes
      2. No
   2. Lung exams
      1. Yes
      2. No
   3. Gastric exams
      1. Yes
      2. No
   4. FAST exams
      1. Yes
      2. No
   5. Airway exams
      1. Yes
      2. No
10. How many minimum scans are required for heart exams?
11. How many minimum scans are required for gastric exams?
12. How many minimum scans are required for FAST exams?
13. How many minimum scans are required for airway exams?
14. Is there a formal assessment for competency?
    1. Yes
    2. No
15. What is the formal assessment based on? (Select all that apply)
16. What percentage of your faculty are trained in POCUS?
    1. 0-25%
    2. 26-50%
    3. 51-75%
    4. 76-100%
17. Is there a faculty POCUS expert in the program?
    1. Yes
    2. No
18. How important is training and competency in the following POCUS exams in modern clinical practice?
    1. Heart
       1. Not at all important
       2. Slightly important
       3. Moderately important
       4. Very important
       5. Extremely important
    2. Lung
       1. Not at all important
       2. Slightly important
       3. Moderately important
       4. Very important
       5. Extremely important
    3. Gastric
       1. Not at all important
       2. Slightly important
       3. Moderately important
       4. Very important
       5. Extremely important
    4. FAST
       1. Not at all important
       2. Slightly important
       3. Moderately important
       4. Very important
       5. Extremely important
    5. Airway
       1. Not at all important
       2. Slightly important
       3. Moderately important
       4. Very important
       5. Extremely important
19. Are there other exams you believe to be important for a graduating anesthesiology resident?
    1. Yes
    2. No
20. What other exams do you believe to be important for a graduating anesthesiology resident?
21. Do you believe a national curriculum should require competency in various POCUS applications? (i.e. heart, lung, gastric, FAST, airway)
    1. Yes
    2. No
22. What various POCUS applications do you believe should be included on a national curriculum? (Please select all that apply)
    1. Heart
    2. Lung
    3. Gastric
    4. FAST
    5. Airway
    6. Others
23. With the integration of POCUS heart (2022), lung (2023), and abdominal (2024) exams into the American Board of Anesthesiology OSCE assessment, what steps is your program taking to improve resident POCUS training?
24. Do you believe the following are barriers to POCUS training at your institution?
    1. Limited machines
       1. Not a barrier
       2. Somewhat a barrier
       3. Moderate barrier
       4. Somewhat significant barrier
       5. Extremely significant barrier
    2. Lack of instructional resources available (i.e. textbooks, online modules, mannequins)
       1. Not a barrier
       2. Somewhat a barrier
       3. Moderate barrier
       4. Somewhat significant barrier
       5. Extremely significant barrier
    3. Lack of funding for extracurricular POCUS training
       1. Not a barrier
       2. Somewhat a barrier
       3. Moderate barrier
       4. Somewhat significant barrier
       5. Extremely significant barrier
    4. Lack of trained staff for formal supervision
       1. Not a barrier
       2. Somewhat a barrier
       3. Moderate barrier
       4. Somewhat significant barrier
       5. Extremely significant barrier
    5. Lack of time during patient care for staff to teach
       1. Not a barrier
       2. Somewhat a barrier
       3. Moderate barrier
       4. Somewhat significant barrier
       5. Extremely significant barrier
    6. Lack of time during patient care for residents to perform POCUS exams
       1. Not a barrier
       2. Somewhat a barrier
       3. Moderate barrier
       4. Somewhat significant barrier
       5. Extremely significant barrier
    7. Lack of formalized POCUS rotation available
       1. Not a barrier
       2. Somewhat a barrier
       3. Moderate barrier
       4. Somewhat significant barrier
       5. Extremely significant barrier
    8. Perceived lack of interest from residents
       1. Not a barrier
       2. Somewhat a barrier
       3. Moderate barrier
       4. Somewhat significant barrier
       5. Extremely significant barrier
25. Are there any other barriers to POCUS training can you think of?
